# Temporal dynamics of the plasma microbiome in recipients at early post-liver transplantation: a retrospective study

**DOI:** 10.1186/s12866-021-02154-w

**Published:** 2021-04-06

**Authors:** Toshihiko Okumura, Kazuhiro Horiba, Hideya Kamei, Suguru Takeuchi, Takako Suzuki, Yuka Torii, Jun-ichi Kawada, Yoshiyuki Takahashi, Yasuhiro Ogura, Tomoo Ogi, Yoshinori Ito

**Affiliations:** 1 Department of Pediatrics, Nagoya University Graduate School of Medicine, 65 Tsurumai-cho, Showa-ku, Nagoya, 466-8550 Japan; 2 Department of Genetics, Research Institute of Environmental Medicine, Nagoya University, Nagoya, Japan; 3 Department of Human Genetics and Molecular Biology, Nagoya University Graduate School of Medicine, Nagoya, Japan; 4 Department of Transplantation Surgery, Nagoya University Hospital, Nagoya, Japan

**Keywords:** Acute cellular rejection, *Enterobacteriaceae*, Liver transplantation, Next-generation sequencing, Plasma microbiome

## Abstract

**Background:**

Immunosuppression during liver transplantation (LT) enables the prevention and treatment of organ rejection but poses a risk for severe infectious diseases. Immune modulation and antimicrobials affect the plasma microbiome. Thus, determining the impact of immunosuppression on the microbiome may be important to understand immunocompetence, elucidate the source of infection, and predict the risk of infection in LT recipients. We characterized the plasma microbiome of LT recipients at early post-LT and assessed the association between the microbiome and clinical events.

**Results:**

In this study, 51 patients who received LT at Nagoya University Hospital from 2016 to 2018 were enrolled. Plasma samples were retrospectively collected at the following time points: 1) within a week after LT; 2) 4 ± 1 weeks after LT; 3) 8 ± 1 weeks after LT; and 4) within 2 days after a positive blood culture. A total of 111 plasma samples were analyzed using shotgun next-generation sequencing (NGS) with the PATHDET pipeline. Relative abundance of *Anelloviridae*, *Nocardiaceae*, *Microbacteriaceae*, and *Enterobacteriaceae* significantly changed during the postoperative period. Microbiome diversity was higher within a week after LT than that at 8 weeks after LT. Antimicrobials were significantly associated with the microbiome of LT recipients. In addition, the proportion of *Enterobacteriaceae* was significantly increased and the plasma microbiome diversity was significantly lower in patients with acute cellular rejection (ACR) than non-ACR patients. Sequencing reads of bacteria isolated from blood cultures were predominantly identified by NGS in 8 of 16 samples, and human herpesvirus 6 was detected as a causative pathogen in one recipient with severe clinical condition.

**Conclusions:**

The metagenomic NGS technique has great potential in revealing the plasma microbiome and is useful as a comprehensive diagnostic procedure in clinical settings. Temporal dynamics of specific microorganisms may be used as indirect markers for the determination of immunocompetence and ACR in LT recipients.

**Supplementary Information:**

The online version contains supplementary material available at 10.1186/s12866-021-02154-w.

## Background

Liver transplantation (LT) is a fundamental treatment for patients with end-stage liver diseases. Immunosuppressive medications after LT enable the prevention and treatment of organ rejection but pose a risk for contraction of severe infectious diseases. Therefore, antimicrobials are often required for patients after LT. Immune modulation and antiviral therapies after organ transplantation are known to affect the gut microbiome [1]. In addition, the viral composition of the plasma microbiome after organ transplantation was reported to be affected by antivirals and immunosuppression [2]. Recently, the assessment of association between the microbiome and clinical events is becoming a major research area. However, few studies have evaluated the plasma microbiome, which comprises bacteria, viruses, and fungi, after transplantation.

Next-generation sequencing (NGS) can comprehensively analyze the sequence data of nucleic acids within a sample. Shotgun metagenomic sequencing allows us to obtain all genes in all organisms present in a clinical sample. In the field of clinical microbiology, NGS has enabled us to detect causative pathogens in various infectious diseases [3–6]. The association of the gut microbiome and its effect on pathophysiology of a number of diseases, for example, cardiac failure, cancer, and liver diseases, have been reported [7–10]. In addition to the gut microbiome, multiple microorganism genomes have been detected in blood, which is essentially sterile [11, 12]. Here, we focused on the plasma microbiome and anticipated that it would be useful to predict clinical adverse events after LT, such as acute cellular rejection (ACR) and infectious diseases. Therefore, in this study, we characterized the plasma microbiome of LT recipients. Temporal dynamics of the plasma microbiome in patients who experienced ACR were also investigated. Moreover, shotgun metagenomic sequencing was evaluated as a detection tool of causative pathogens.

## Results

### Temporal dynamics of the plasma microbiome

A total of 111 plasma samples were consecutively collected from 51 LT recipients. First, we analyzed 25 recipients whose samples had been collected at all three time points. Subsequently, 28 samples from other patients were analyzed to confirm the results. We obtained an average of 13.5 million reads per sample. Approximately 0.03% of the total reads were identified as microorganism reads after subtraction of human-derived reads and artifacts (Additional file 1).

**Fig. 1:**
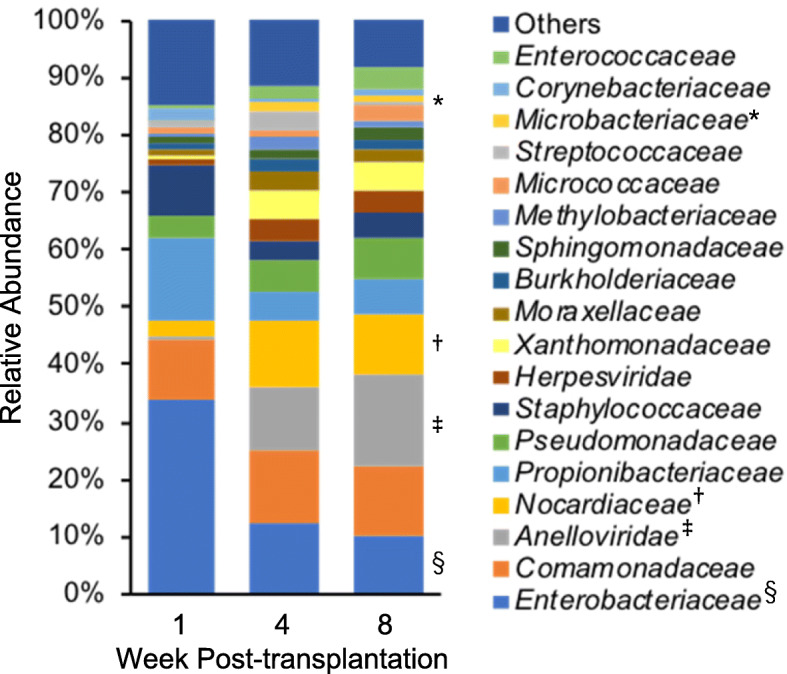
Change in the relative abundance of each microorganism at the family level in plasma samples after liver transplantation. *Microbacteriaceae* (*), *Nocardiaceae* (†), and *Anelloviridae* (‡) abundance increased in samples collected at week 4 or 8 post-transplantation in comparison with samples collected at week 1 post-transplantation (*Anelloviridae*: week 4 vs. week 1, *p* = 0.62, week 8 vs. week 1, *p* < 0.001; *Nocardiaceae*: week 4 vs. week 1, *p* = 0.003, week 8 vs. week 1, *p* = 0.36; *Microbacteriaceae*: week 4 vs. week 1, *p* < 0.001, week 8 vs. week 1, *p* = 0.002). *Enterobacteriaceae* (§) abundance decreased in samples collected at week 4 or 8 post-transplantation in comparison with samples collected at week 1 post-transplantation (week 4 vs. week 1, *p* < 0.001, week 8 vs. week 1, *p* < 0.001)

**Fig. 2:**
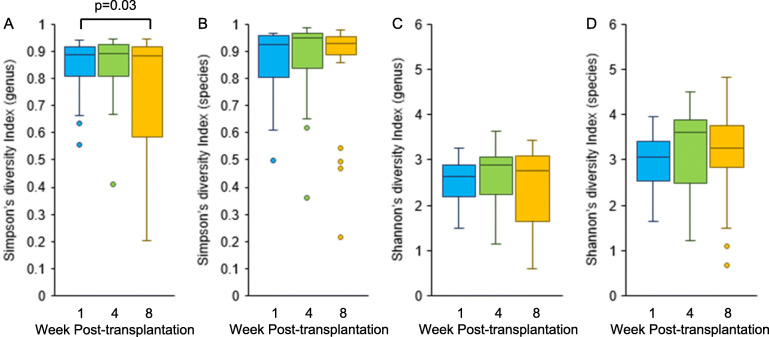
Change in plasma microbiome diversity at genus and species levels after liver transplantation. **a** Simpson’s diversity index at the genus level, **b** Simpson’s diversity index at the species level, **c** Shannon’s diversity index at the genus level, and **d** Shannon’s diversity index at the species level at each sampling point are shown. Simpson’s diversity index (at the genus level) of samples collected at week 1 post-transplantation was significantly higher than that of samples collected at week 8 post-transplantation (0.85 ± 0.09 vs. 0.76 ± 0.23, respectively, *p* = 0.03)

Figure 1 shows the time-dependent relative composition of plasma microorganisms. Data include all samples (46 samples collected at week 1, 28 samples collected at week 4, and 29 samples collected at week 8 post-transplantation) and show average relative abundance at the family level of taxonomic hierarchy. *Anelloviridae*, *Nocardiaceae*, and *Microbacteriaceae* were significantly increased in samples collected at week 4 or 8 post-transplantation compared with those collected at week 1 post-transplantation (*Anelloviridae*: 0.07 ± 0.19 (week 4) vs. 0.01 ± 0.04 (week 1), *p* = 0.62, 0.23 ± 0.28 (week 8) vs. 0.01 ± 0.04 (week 1), *p* < 0.001; *Nocardiaceae*: 0.127 ± 0.100 (week 4) vs. 0.057 ± 0.084 (week 1), *p* = 0.003, 0.089 ± 0.074 (week 8) vs. 0.057 ± 0.084 (week 1), *p* = 0.36; *Microbacteriaceae*: 0.017 ± 0.013 (week 4) vs. 0.004 ± 0.007 (week 1), *p* < 0.001, 0.013 ± 0.012 (week 8) vs. 0.004 ± 0.007 (week 1), *p* = 0.002) whereas *Enterobacteriaceae* was decreased (0.13 ± 0.12 (week 4) vs. 0.26 ± 0.17 (week 1), *p* < 0.001, 0.09 ± 0.11 (week 8) vs. 0.26 ± 0.17 (week 1), *p* < 0.001). The time-dependent relative compositions of plasma microorganisms at the genus and species levels are shown in Figs. S1 and S2. Figure 2 shows the temporal changes in plasma microbiome diversity. Simpson’s diversity index at the genus level at week 1 post-transplantation was significantly higher than that at week 8 post-transplantation (0.85 ± 0.09 vs. 0.76 ± 0.23, respectively, *p* = 0.03). There was no difference in Shannon’s diversity index during the postoperative period.

### Association between the plasma microbiome and clinical variations

**Fig. 3:**
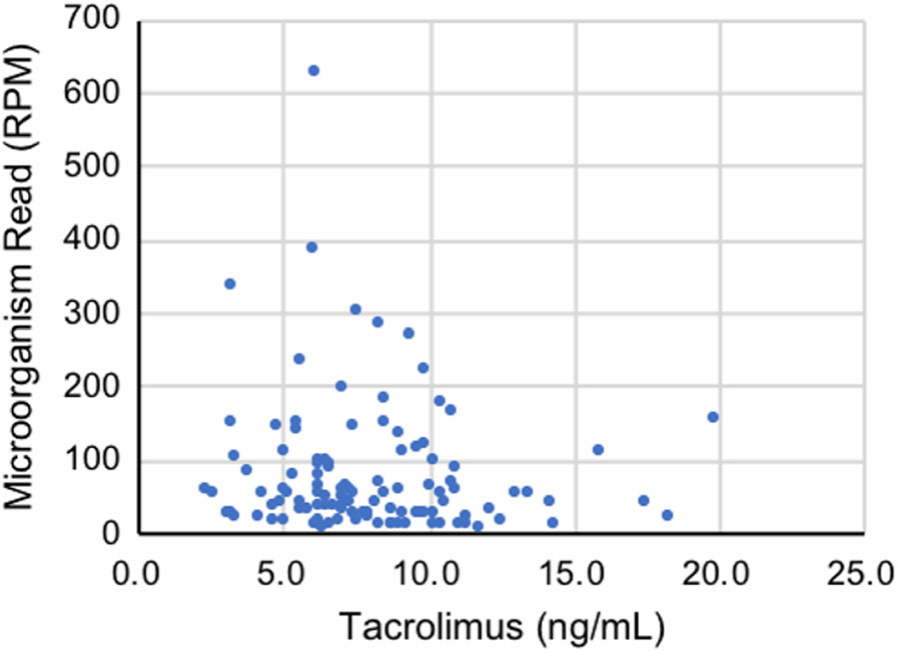
Correlation between the number of microorganism reads and the tacrolimus concentration after liver transplantation. The number of microorganism reads and the tacrolimus concentration after liver transplantation are plotted. There was no significant correlation between the two indicators (r = 0.05, *p* = 0.62). RPM, reads per million mapped reads

The post-transplant plasma microbiome and immunosuppressive medications were also investigated. There was no significant correlation between microorganism reads and tacrolimus concentration at the time of sample collection (Fig. 3). In addition, no significant correlation was detected between each microorganism and tacrolimus concentration (Additional file 2).

**Fig. 4:**
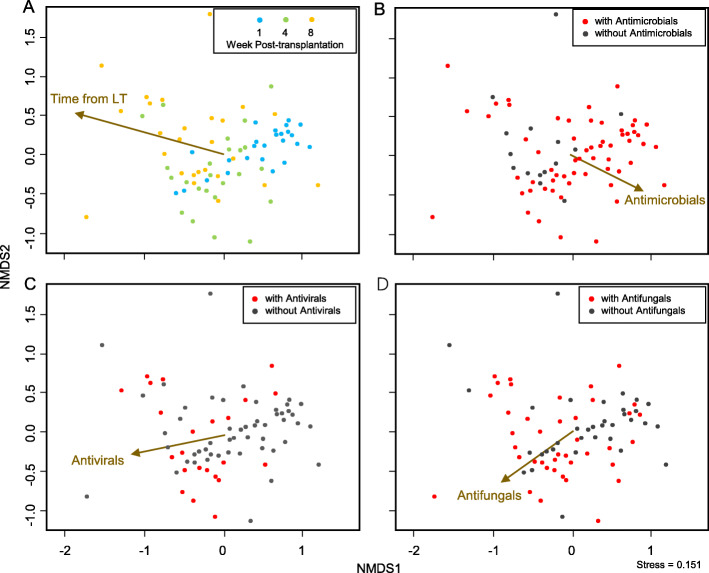
Non-metric multidimensional scaling representation of plasma samples at each sampling point with envfit correlations. Samples were plotted based on Bray–Curtis distances calculated using family compositions (stress value = 0.151). Arrows represent envfit correlations for **a** time from LT, **b** antimicrobials, **c** antivirals, and **d** antifungals; strong factors have longer arrows, whereas weak factors have shorter arrows. LT, liver transplantation; NMDS, non-metric multidimensional scaling

We obtained the microbiome of 75 samples after evaluating the quality of sequencing data using rarefaction curves. The non-metric multidimensional scaling (NMDS) plot of the microbiome of 75 samples showed that they tended to cluster together per time point (Fig. 4a). The analysis of reads per million mapped reads (RPM) profiles and clinical information (age, time point, tacrolimus, mycophenolate mofetil, steroids, antibacterial, antiviral, and antifungal) at the time of sample collection revealed that antimicrobials were associated with the microbiome. Samples collected after the administration of each antimicrobial tended to lean in the direction of each arrow (Fig. 4b-d). By contrast, age and immunosuppressive medications did not have significant correlations with the microbiome.

When patients were divided into two groups, children (*n* = 30) and adults (*n* = 81), there were some differences in individual microorganisms. At the family level, Enterobacteriaceae and *Propionibacteriaceae* were increased in the children group, whereas *Anelloviridae*, *Nocardiaceae*, and *Methylobacteriaceae* were increased in the adults group (*Enterobacteriaceae*: 0.24 ± 0.16 vs. 0.15 ± 0.15, respectively, *p* = 0.015; *Propionibacteriaceae*: 0.11 ± 0.10 vs. 0.07 ± 0.07, respectively, *p* = 0.012; *Anelloviridae*: 0.01 ± 0.03 vs. 0.11 ± 0.22, respectively, *p* < 0.001; *Nocardiaceae*: 0.05 ± 0.06 vs. 0.09 ± 0.10, respectively, *p* = 0.012; *Methylobacteriaceae*: 0.01 ± 0.02 vs. 0.02 ± 0.03 respectively, *p* = 0.011; Fig. S3).

### Association between the plasma microbiome and ACR

**Fig. 5:**
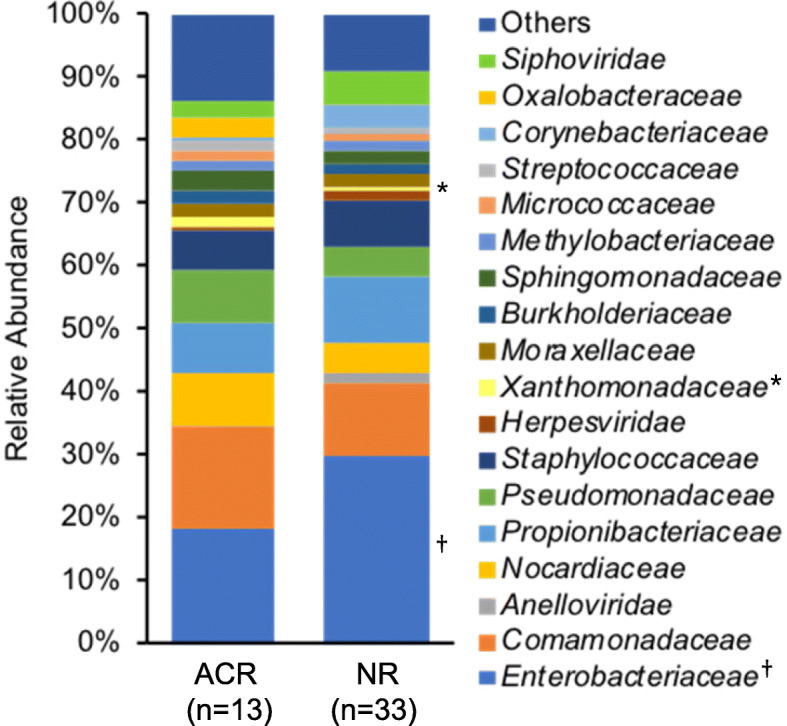
Comparison of the plasma microbiome at the family level in patients with and without ACR. *Xanthomonadaceae* (*) abundance was significantly increased in the ACR group in comparison with the no rejection (NR) group (*p* = 0.047). In contrast, *Enterobacteriaceae* (†) abundance was significantly decreased in the ACR group in comparison with the NR group (*p* = 0.045). ACR, acute cellular rejection; NR, no rejection

**Fig. 6:**
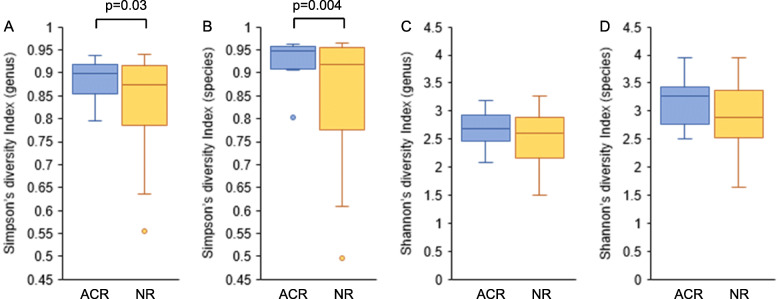
Comparison of plasma microbiome diversity at genus and species levels in patients with and without ACR. **a** Simpson’s diversity index at the genus level, **b** Simpson’s diversity index at the species level, **c** Shannon’s diversity index at the genus level, and **d** Shannon’s diversity index at the species level are depicted. Simpson’s diversity indices in the ACR group were significantly higher than those in the NR group (genus level: 0.89 ± 0.04 vs. 0.84 ± 0.10, respectively, *p* = 0.03; species level: 0.93 ± 0.04 vs. 0.85 ± 0.13, respectively, *p* = 0.004). ACR, acute cellular rejection; NR, no rejection

Forty-six patients, whose samples were collected at week 1 post-transplantation, were included in the analysis to assess the association between the plasma microbiome and ACR. Table 1 shows the characteristics of patients in ACR and “no rejection” (NR) groups. Adult patients in the ACR group were significantly younger than those in the NR group (*p* = 0.02). The mean tacrolimus concentrations that had been examined before the development of ACR and in the first 2 weeks after LT in the NR group showed no significant difference. Comparing the average relative abundance at the family level, *Xanthomonadaceae* was increased in the ACR group, whereas *Enterobacteriaceae* was increased in the NR group (*Xanthomonadaceae*: 0.015 ± 0.015 vs. 0.005 ± 0.007, respectively, *p* = 0.047; *Enterobacteriaceae*: 0.18 ± 0.16, vs. 0.30 ± 0.17, respectively, *p* = 0.045; Fig. 5). Comamonadaceae and Nocardiaceae abundance tended to be higher in the ACR group than in the NR group, but there was no significant correlation. As shown in Fig. 6, Simpson’s diversity indices in the ACR group were significantly higher than those in the NR group (genus level: 0.89 ± 0.04 vs. 0.84 ± 0.10, respectively, *p* = 0.03; species level: 0.93 ± 0.04 vs. 0.85 ± 0.13, respectively, *p* = 0.004).

**Table 1:** Patient characteristics and comparisons between acute cellular rejection (ACR) and no rejection (NR) groups

	ACR (*n* = 13)	NR (*n* = 33)	*p*-value
No. patients (pediatric/adult)^a^	4/9	8/25	0.72
Age at LT [median (range)]			
Pediatric	13 m (5–50 m)	10.5 m (7–25 m)	0.93
Adult	39 y (18–63 y)	54 y (24–66 y)	0.02
Sex (male/female)	5/8	11/22	0.74
Living donor/brain death donor	11/2	25/8	0.70
ABO incompatible	3 (23.1%)	4 (12.1%)	0.39
Tacrolimus concentrations (mean ± SD)	7.7 ± 1.5	8.0 ± 1.3	0.59
AST (mean ± SD) ^b^	197 ± 155	235 ± 406	0.75
ALT (mean ± SD) ^b^	409 ± 295	318 ± 366	0.43
Initially treated with mycophenolate mofetil	9 (69.2%)	20 (60.6%)	0.74
Underlying disease			
Biliary atresia	4 (30.8%)	6 (18.2%)	0.44
Primary sclerosing cholangitis	2 (15.4%)	6 (18.2%)	1.00
Primary biliary cholangitis	2 (15.4%)	3 (9.1%)	0.61
Fulminant hepatic failure	1 (7.7%)	4 (12.1%)	1.00
Hepatitis B	2 (15.4%)	2 (6.1%)	0.57
Hepatitis C	1 (7.7%)	3 (9.1%)	1.00
Autoimmune hepatitis	1 (7.7%)	2 (6.1%)	1.00
Others	2 (15.4%)	7 (21.2%)	
Days of hospitalization after LT [median (range)]	86 (37–208)	59 (24–326)	0.25

NOTE: Data are given as n (%) unless otherwise noted. ^a^One patient with hepatitis B developed fulminant hepatic failure, and another patient with hepatitis B developed primary biliary cholangitis in the ACR group. ^b^The peak levels of transaminases observed between days 4 and 14 post-transplantation. *P*-values were calculated by the Mann–Whitney *U* test or Fisher’s exact test. *LT* Liver transplantation, *AST* Aspartate aminotransferase, *ALT* Alanine aminotransferase

### Comparison of NGS results in plasma samples with blood culture results

**Fig. 7:**
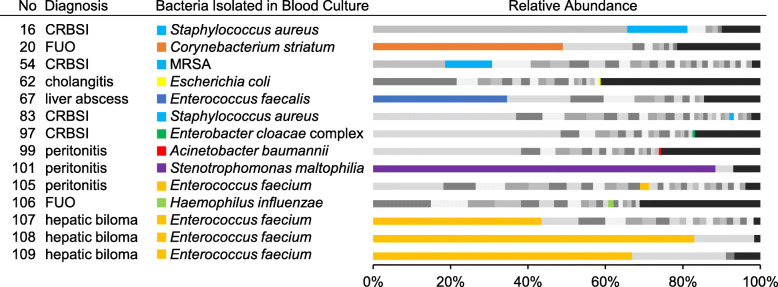
Relative abundance of microorganisms at the species level in plasma from patients with positive blood cultures. Representative results of 14 patients are shown. Colored bars indicate that the same bacteria were isolated from next-generation sequencing and blood culture samples. Black bars indicate microorganisms with < 1% relative abundance. CRBSI, catheter-related bloodstream infection; FUO, fever of undetermined origin; MRSA, methicillin-resistant *Staphylococcus aureus*

Sixteen samples were collected within 2 days after positive blood culture. The same bacterial species isolated in blood culture were identified by NGS in 14 of 16 samples, and predominant pathogens in blood culture were identified in 8 samples (Nos. 16, 20, 54, 67, 101, 107, 108, and 109; Fig. 7). Of note, the microorganism that accounted for the highest relative abundance in sample No. 16 was human herpesvirus 6 (HHV-6) and that in sample No. 54 was *Propionibacterium* sp. oral taxon 193. Moreover, sample Nos. 107, 108, and 109 were obtained from the same patient who continuously suffered from bloodstream infection for over a month.

Given the days of interval between NGS sample collection and blood culture sample collection, predominant bacterial species were identical in 4 of 6 recipients when the two samples were collected on the same day. Conversely, predominant bacterial species were identical in 4 of 10 samples when the interval of sample collection was 1–2 days apart. Although we checked several samples collected a few days before blood culture collection, the same predominant bacteria were not identified by NGS.

An overwhelming amount of HHV-6 was detected in sample No. 111 (relative abundance = 99.9%) with negative blood culture. The sample was collected from a 42-year-old female who received LT for drug-induced acute liver failure. Due to vascular complications, she had a complicated postoperative course with rapid elevation of liver enzymes. Unfortunately, she died after a week despite intensive care.

## Discussion

In this study, we examined the plasma microbiome of LT recipients using NGS. Majority of the bacteria are separated out by centrifugation during plasma prepping; however, several studies have examined the microbiome in plasma, which we believe is a well-established method [13, 14]. In this study, 95.18–99.97% of the available reads were of human origin, even from plasma samples, and deep sequencing by NGS enabled detection of the microorganism genome in the plasma samples. Most of the microorganisms that we detected may be their DNA constituents in the blood, except for bacteremia, because NGS can capture all genes in the samples if they are fragmented. The sources of these microorganisms are considered to be the gut or oral mucosa, from where they invade the blood [15, 16]. Other sources may be contamination by normal skin-inhabiting microorganisms or even reagent contamination.

The plasma microbiome clearly changed from immediately after LT to 8 weeks post-transplantation. Interestingly, *Anelloviridae* and *Enterobacteriaceae* abundance dynamically changed during this period. The relative abundance of *Anelloviridae* increased and that of *Enterobacteriaceae* decreased in the postoperative course. The same trends were observed at the genus and species levels, but the amounts of microorganisms belonging to the genus and species were too small to identify individual microorganisms. *Anelloviridae* mainly consists of Torque teno virus (TTV). TTV, a small non-enveloped single-stranded DNA virus, is extremely common in the human population, and TTV DNA in plasma can be detected in about 75% of healthy individuals [17, 18]. It is considered that TTV is not associated with any specific human illness at present [19]. Maggi et al. reported that TTV load in plasma from patients who received kidney or liver transplantation increased after transplantation [20]. Other studies also reported that TTV load in plasma or bronchoalveolar lavage from patients who received lung transplantation increased after transplantation [21, 22]. In the present study, *Anelloviridae* was scarcely detected in samples immediately after LT and significantly increased several weeks later. This change indicated that *Anelloviridae* abundance increased under immunosuppressive therapy at a higher rate than other microorganisms. Although we expected that the number of microorganism reads would increase if immunosuppression therapy was strong, there was no significant correlation between the number of pathogen reads and tacrolimus concentration. In addition, there was no significant correlation between each microorganism and tacrolimus concentration in the present study. One possible reason is that tacrolimus concentration alone was insufficient to measure the degree of immunosuppression because immunosuppressive medication comprises not only tacrolimus but also mycophenolate mofetil and steroids. Comparing children and adults, they have different microbiomes, even in healthy individuals [23]. Furthermore, the underlying diseases were very different in the present study. In particular, children are rarely infected with *Anelloviridae,* which is rarely detected.

The NMDS plot of the microbiome showed high similarity of the microbiome among samples at the same time point. As antibiotics were administered prophylactically after operation, samples collected at week 1 post-transplantation would be classified as samples with antibiotics. Antifungals and antivirals were also associated with the microbiome. De Vlaminck et al. reported that dose of immunosuppressants and antivirals affect the viral component in plasma and the virome changes during the post-transplantation period. *Herpesvirales* and *Caudovirales* dominated the virome when patients received low doses of these drugs, and *Anelloviridae* dominated the virome when patients received high doses of these drugs [2]. Antibiotic treatment, infection, and LT itself were reported to be factors that change the structure of the bacterial component of the gut microbiome [24–27]. Our results showed that antimicrobial therapy was correlated with the plasma microbiome, but immunosuppressive medications were not. The change in the plasma microbiome may not be only due to the effect of antimicrobials, but also other factors under severe conditions in LT recipients.

In terms of ACR, patients in the NR group were expected to be more immunosuppressed in comparison with the ACR group because ACR indicates the development of strong immune responses. Comparison of the microbiome during the postoperative period indicated that samples collected at week 1 post-transplantation under strong immunosuppression had high relative abundance of *Enterobacteriaceae*. The increase in relative abundance of *Enterobacteriaceae* may reflect the strength of immunosuppression. Previous studies reported that abundance of potentially pathogenic *Enterobacteriaceae* in gut microbiota increased in patients after LT [1, 28], and bacteria constituting the gut microbiota caused bloodstream infections [15]. *Enterobacteriaceae* detected in plasma was also considered to originate from the gut microbiota in the present study. No studies have hitherto referred to the link between ACR and leaky gut syndrome, but considering the results of this study, it is possible that *Enterobacteriaceae* easily invade the bloodstream from the gut in patients under strong immunosuppression. Moreover, Simpson’s diversity indices in the NR group were lower than those in the ACR group. This result might be explained by the finding that *Enterobacteriaceae* accounted for nearly 30% of plasma microorganisms in the NR group. *Enterobacteriaceae* genome in plasma and the high diversity of the plasma microbiome may be a biomarker for the development of ACR after LT. *Xanthomonadaceae* abundance was increased in the ACR group, but its association with ACR was unclear because the amount of *Xanthomonadaceae* detected in this study was small. The causes and results of changes in the microbiome and the development of ACR are also unclear. If the microbiome is the cause, fecal microbiota transplantation or probiotics may prevent ACR, but there are no reports of intervention at this time.

In this study, bacterial species identical to those isolated by blood culture were predominantly detected by NGS in 8 of 16 samples. The detection rate for bacterial species by NGS was comparable to rates reported by previous studies (50–70%) [29–31]. However, detection rates of samples collected after sampling for blood cultures were rather low (4 of 10, 40%) in this study. Bacteria were not detected by NGS in the samples collected before sampling for blood cultures, suggesting that the prediction of bloodstream infections by NGS is difficult. Previous studies [29–31] reported that pathogens could be detected by NGS in about 20% of culture-negative samples. Of note, an overwhelming abundance of HHV-6 was detected in one patient when the patient’s condition clinically worsened. NGS is expected to be a comprehensive diagnostic tool for pathogens in the clinical setting.

This study has several limitations. First, as this was a retrospective study, the details of blood sample collection were not available. Therefore, contamination cannot be excluded. Second, the recipients were heterogenous, particularly including adult and pediatric recipients. Third, there were no control samples before LT. Thus, the plasma microbiomes before and after LT were not compared. Whether the plasma microbiome after LT changes to the microbiome before LT is of great interest. Moreover, control samples from healthy individuals are necessary to know whether the microbiome of LT recipients becomes similar to that of healthy individuals.

## Conclusions

In conclusion, the plasma microbiome and temporal dynamics in LT recipients were analyzed using NGS. *Anelloviridae* and *Enterobacteriaceae* abundance dynamically changed in the early phase of post-transplantation. The NMDS analysis revealed that antimicrobials are associated with the microbiome composition. Importantly, *Enterobacteriaceae* abundance and high plasma microbiome diversity may be indicators for the development of ACR after LT. NGS is useful as a comprehensive diagnostic tool for pathogens in LT recipients.

## Methods

### Patients and samples

Fifty-one patients who received LT at Nagoya University Hospital between 2016 and 2018 were enrolled in this study. Inclusion criteria were the availability of a plasma sample within a week after LT (presented as the sample collected at week 1 post-transplantation) or within 2 days after a positive blood culture. For some patients, we followed plasma samples 4 ± 1 weeks after LT (presented as the sample collected at week 4 post-transplantation) and 8 ± 1 weeks after LT (presented as the sample collected at week 8 post-transplantation). Table 2 summarizes the characteristics of the 51 patients (13 children and 38 adults). Underlying diseases for LT were biliary atresia in 10 patients followed by primary sclerosing cholangitis in 9 patients, primary biliary cholangitis in 6 patients, and fulminant hepatic failure in 6 patients.

**Table 2:** Demographic characteristics of LT recipients

	Total (*n* = 51)
No. patients (pediatric/adult)^a^	13/38
Age at LT [median (range)]	
Pediatric	12 m (5–50 m)
Adult	50.5 y (18–66 y)
Sex (male/female)	19/32
Living donor/brain death donor	38/13
ABO-incompatible	8 (15.7%)
Underlying disease	
Biliary atresia	10 (19.6%)
Primary sclerosing cholangitis	9 (17.6%)
Primary biliary cholangitis	6 (11.8%)
Fulminant hepatic failure	6 (11.8%)
Hepatitis B	4 (7.8%)
Hepatitis C	4 (7.8%)
Autoimmune hepatitis	3 (5.9%)
Others	11 (21.6%)
ACR	19 (37.3%)
Days from LT to ACR [median (range)]	8 (4–56)
Days of hospitalization after LT [median (range)]	62 (24–326)

NOTE: Data are given as n (%) unless otherwise noted. ^a^One patient with hepatitis B developed fulminant hepatic failure, and another patient with hepatitis B developed primary biliary cholangitis. *LT* Liver transplantation, *ACR* Acute cellular rejection

ACR was defined as abnormal findings of liver tissue that indicated acute rejection through a biopsy performed on patients with clinical suspicion, such as fever and hepatic dysfunction, within 3 months after LT. We defined the ACR group as patients who developed ACR within 2 weeks after LT (13 of 19 patients with ACR) to evaluate the association between ACR and plasma microbiome at week 1 post-transplantation. The patient group without rejection was defined as patients with NR.

The median number of hospitalization days after LT was 62 days (range, 24–326 days). Immunosuppressive medications including tacrolimus and steroids were administered to all patients after LT. The target trough levels of tacrolimus were 10–15 ng/mL for the first 2 weeks, 5–10 ng/mL for the next 2 months, and 4–6 ng/mL thereafter. Methylprednisolone was initially administered intravenously at a dose of 10 mg/kg immediately after reperfusion, followed by 1 mg/kg for the first 3 days, 0.5 mg/kg for the next 3 days, and 0.3 mg/kg on postoperative day 7. Oral prednisolone was subsequently administered at a dose of 0.3 mg/kg once daily and tapered. Patients who received ABO blood-type incompatible transplants or had other indications for maintaining low tacrolimus concentrations, such as renal dysfunction, were treated with mycophenolate mofetil. Furthermore, adult patients who received ABO blood-type incompatible transplants were treated with rituximab and plasma exchange preoperatively and received additional methylprednisolone via the portal vein postoperatively. All patients received antibiotic prophylaxis with cefotaxime and ampicillin for 48 h postoperatively. Antifungal and antiviral prophylaxes were not routinely administered.

### Sample preparation and sequencing

Collected plasma samples were stored at − 30 °C until use. DNA was extracted from 140-μL plasma samples using the QIAamp UCP Pathogen Mini Kit (Qiagen, Hilden, Germany). DNA concentrations were measured using the Qubit dsDNA HS Assay Kit (Thermo Fisher Scientific, Waltham, MA, USA). DNA sequencing libraries were prepared using the Nextera XT Library Prep Kit (Illumina, San Diego, CA, USA) according to the manufacturer’s instructions with slight modifications to increase the library concentration: in the clean up step, DNA libraries were purified twice using AMPure XP beads and final elution was carried out in 42.5 μL of resuspension buffer. The library quality was analyzed using the Agilent 2100 bioanalyzer (Agilent, Santa Clara, CA, USA) and droplet digital PCR. DNA libraries were sequenced on the HiSeq system (Illumina) with the 2 × 150 bp paired-end protocol in the rapid run mode.

### NGS data processing

To detect microbial sequences in plasma samples, NGS data files were imported into a newly developed bioinformatic pipeline named PATHDET. PATHDET is published on the internet and is free to use (PATHDET v1.0, https://pathdet.hgc.jp/). RPM of each microorganism were calculated in each plasma sample as the number of microorganism sequence reads per million sequence reads. Relative abundance was also calculated as the percentage of microorganism sequence reads in the total microorganism reads. Additionally, Simpson’s diversity index and Shannon’s diversity index based on each taxonomic hierarchy were calculated from the microorganism reads.

Next, we constructed a NMDS plot of microorganism beta-diversities measured by Bray–Curtis dissimilarity between RPM profiles at the family level using the R package vegan (https://CRAN.R-project.org/package=vegan). NMDS was used to visualize the level of similarity of the microbiome by considering dissimilarity distance matrices among samples. Before constructing the plot, we computed rarefaction curves to evaluate the quality of sequencing data. To identify factors that affect the microbiome, we compared RPM profiles with clinical information (age, time point, tacrolimus, mycophenolate mofetil, steroids, antibacterial, antiviral, and antifungal) at the time of sample collection using the function envfit. The envfit function fits environmental factors onto the ordination, with the length of the arrow proportional to the correlation with the ordination.

### Statistical analysis

Continuous variables are presented as the median (range). Results were compared using Fisher’s exact tests for categorical variables and the Mann–Whitney *U* test for continuous variables. Index values, RPM, relative abundance, and each diversity index among the three time points were analyzed by analysis of variance and post hoc tests were conducted using the Bonferroni test. Correlations between RPM and tacrolimus concentration were performed using Pearson’s correlation analysis and correlation coefficients (r) were reported. Index values between ACR and NR groups were compared using Student’s *t*-test. SPSS version 25.0 (IBM, Chicago, IL, USA) was used to perform the statistical analyses, and *p*-values < 0.05, were considered statistically significant.

## Supplementary Information

**Additional file 1.** The number of reads in each sample.

**Additional file 2.** Correlation between each microorganism and tacrolimus concentration.

**Additional file 3: Fig. S1** Change in the relative abundance of each microorganism at the genus level in plasma samples after liver transplantation. **Fig.** S2 Change in the relative abundance of each microorganism at the species level in plasma samples after liver transplantation. **Fig. S3** Comparison of the plasma microbiome at the family level in children and adults. *Methylobacteriaceae* (*), *Nocardiaceae* (‡), and *Anelloviridae* (§) abundances were significantly lower in children than in adults (*p* = 0.011, 0.012, and < 0.001, respectively). In contrast, *the abundance of Propionibacteriaceae* (†) and *Enterobacteriaceae* (||) was significantly higher in children than in adults (*p* = 0.012 and 0.015, respectively).

## Data Availability

The datasets used and/or analyzed during the current study are available from the corresponding author on reasonable request.

## Abbreviations

ACR: Acute cellular rejection
CRBSI: Catheter-related bloodstream infection
FUO: Fever of undetermined origin
HHV-6: Human herpesvirus 6
LT: Liver transplantation
MRSA: Methicillin-resistant *Staphylococcus aureus*
NGS: Next-generation sequencing
NMDS: Non-metric multidimensional scaling
NR: No rejection
PCR: Polymerase chain reaction
RA: Relative abundance
RPM: Reads per million mapped reads
TTV: Torque teno virus
