# Supplementary material for: Temporal dynamics of the plasma microbiome in recipients at early post-liver transplantation: a retrospective study
Source: BMC Microbiol. 2021 Apr 6;21:104. doi: 10.1186/s12866-021-02154-w (PMC8025517; doi:10.1186/s12866-021-02154-w)
Supplement: Supplementary file 3 — Additional file 3: Fig. S1 Change in the relative abundance of each microorganism at the genus level in plasma samples after liver transplantation. Fig. S2 Change in the relative abundance of each microorganism at the species level in plasma samples after liver transplantation. Fig. S3 Comparison of the plasma microbiome at the family level in children and adults. Methylobacteriaceae (*), Nocardiaceae (‡), and Anelloviridae (§) abundances were significantly lower in children than in adults (p = 0.011, 0.012, and < 0.001, respectively). In contrast, the abundance of Propionibacteriaceae (†) and Enterobacteriaceae (||) was significantly higher in children than in adults (p = 0.012 and 0.015, respectively). [file 12866_2021_2154_MOESM3_ESM.pptx]

## Slide 1
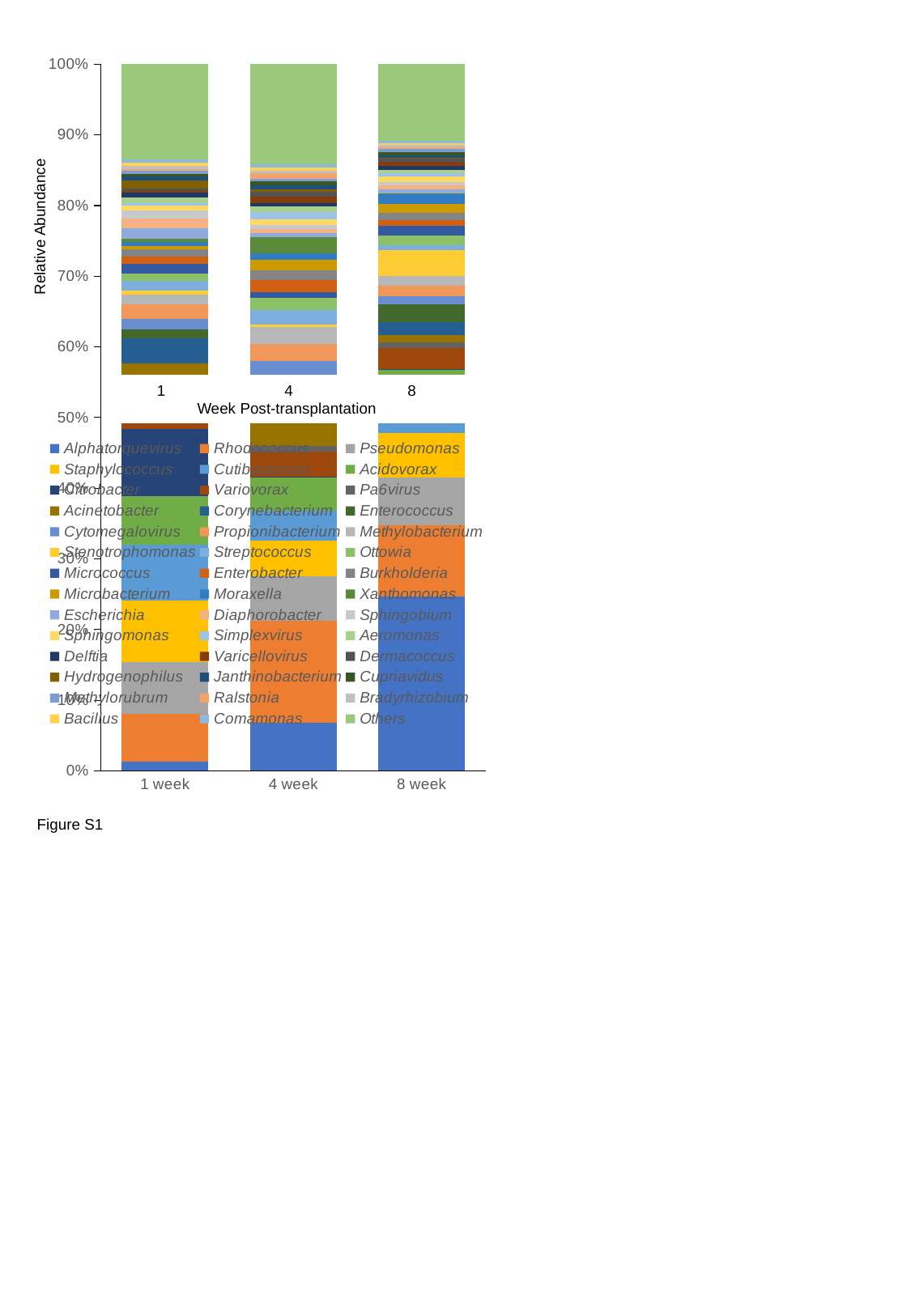

### Chart
| Category | Alphatorquevirus | Rhodococcus | Pseudomonas | Staphylococcus | Cutibacterium | Acidovorax | Citrobacter | Variovorax | Pa6virus | Acinetobacter | Corynebacterium | Enterococcus | Cytomegalovirus | Propionibacterium | Methylobacterium | Stenotrophomonas | Streptococcus | Ottowia | Micrococcus | Enterobacter | Burkholderia | Microbacterium | Moraxella | Xanthomonas | Escherichia | Diaphorobacter | Sphingobium | Sphingomonas | Simplexvirus | Aeromonas | Delftia | Varicellovirus | Dermacoccus | Hydrogenophilus | Janthinobacterium | Cupriavidus | Methylorubrum | Ralstonia | Bradyrhizobium | Bacillus | Comamonas | Others |
|---|---|---|---|---|---|---|---|---|---|---|---|---|---|---|---|---|---|---|---|---|---|---|---|---|---|---|---|---|---|---|---|---|---|---|---|---|---|---|---|---|---|---|
| 1 week | 0.012881220869565217 | 0.06735952680434783 | 0.07390225847826085 | 0.08725397608695654 | 0.07823029434782608 | 0.06878989456521738 | 0.09555830347826087 | 0.02052025895652174 | 0.055145660217391305 | 0.016612536347826088 | 0.036429146956521746 | 0.011615297173913044 | 0.01572445239130435 | 0.02048793554347826 | 0.013285985391304345 | 0.005715788913043479 | 0.014165707826086954 | 0.009957875869565216 | 0.013824113260869565 | 0.010908570608695654 | 0.009757507000000002 | 0.004182078173913044 | 0.00664555847826087 | 0.004349960760869566 | 0.014547617391304347 | 0.013609374130434783 | 0.012191040652173914 | 0.006623663391304347 | 0.003607489282608696 | 0.007083485826086956 | 0.007818683717391306 | 0.0013498695652173912 | 0.0041443455 | 0.011183297391304348 | 0.0055967889565217395 | 0.003869004891304348 | 0.004369648260869565 | 0.002066464695652174 | 0.004516360586956522 | 0.005211918413043477 | 0.003805887260869565 | 0.13510120491304325 |
| 4 week | 0.06759107821428571 | 0.14460788214285714 | 0.06361465000000001 | 0.049733220714285716 | 0.04226764428571428 | 0.04853824214285714 | 0.00029313464285714286 | 0.03498563285714286 | 0.007555261785714286 | 0.04468276 | 0.014037846428571426 | 0.029992364999999997 | 0.032066508571428565 | 0.023850975000000003 | 0.023976843928571433 | 0.0033653164285714285 | 0.020930094285714285 | 0.017417588571428576 | 0.008349187857142858 | 0.017314527857142856 | 0.013637215357142856 | 0.01447570464285714 | 0.009430528214285713 | 0.02296797357142857 | 0.0054647664285714295 | 0.005764695357142856 | 0.005633030357142856 | 0.007595535 | 0.010880607142857143 | 0.0077117367857142865 | 0.004490817142857142 | 0.009678642857142858 | 0.007039145000000001 | 0.0031933256071428567 | 0.005316825 | 0.005446677499999999 | 0.0042273982142857144 | 0.007530845357142857 | 0.003885404642857142 | 0.004094745357142856 | 0.004450220714285713 | 0.14191344014285712 |
| 8 week | 0.2464218444137931 | 0.10097836965517239 | 0.06786609344827588 | 0.06279822655172414 | 0.049764068758620675 | 0.04046639655172414 | 0.0004910134137931035 | 0.029499904482758625 | 0.008430207586206896 | 0.009696405862068967 | 0.019093548965517234 | 0.025227251000000003 | 0.01138837444827586 | 0.014171368620689654 | 0.01463051103448276 | 0.03589946620689655 | 0.006598159310344827 | 0.013753203793103451 | 0.013894823000000002 | 0.007780091379310344 | 0.011311556896551722 | 0.01182214275862069 | 0.013506366551724141 | 0.0016472310344827585 | 0.005421889896551725 | 0.0058868579310344836 | 0.005257086896551724 | 0.007458109310344829 | 0.005135724137931035 | 0.004623193793103449 | 0.005403693689655172 | 0.005898775862068966 | 0.004793438206896551 | 0.0010570431034482761 | 0.00314908748275862 | 0.003967661379310344 | 0.004483914137931034 | 0.002691365068965518 | 0.003483099655172414 | 0.0024674893103448273 | 0.0028949165517241386 | 0.10878986613793105 |Relative Abundance
1 4 8
Week Post-transplantation
Figure S1

## Slide 2
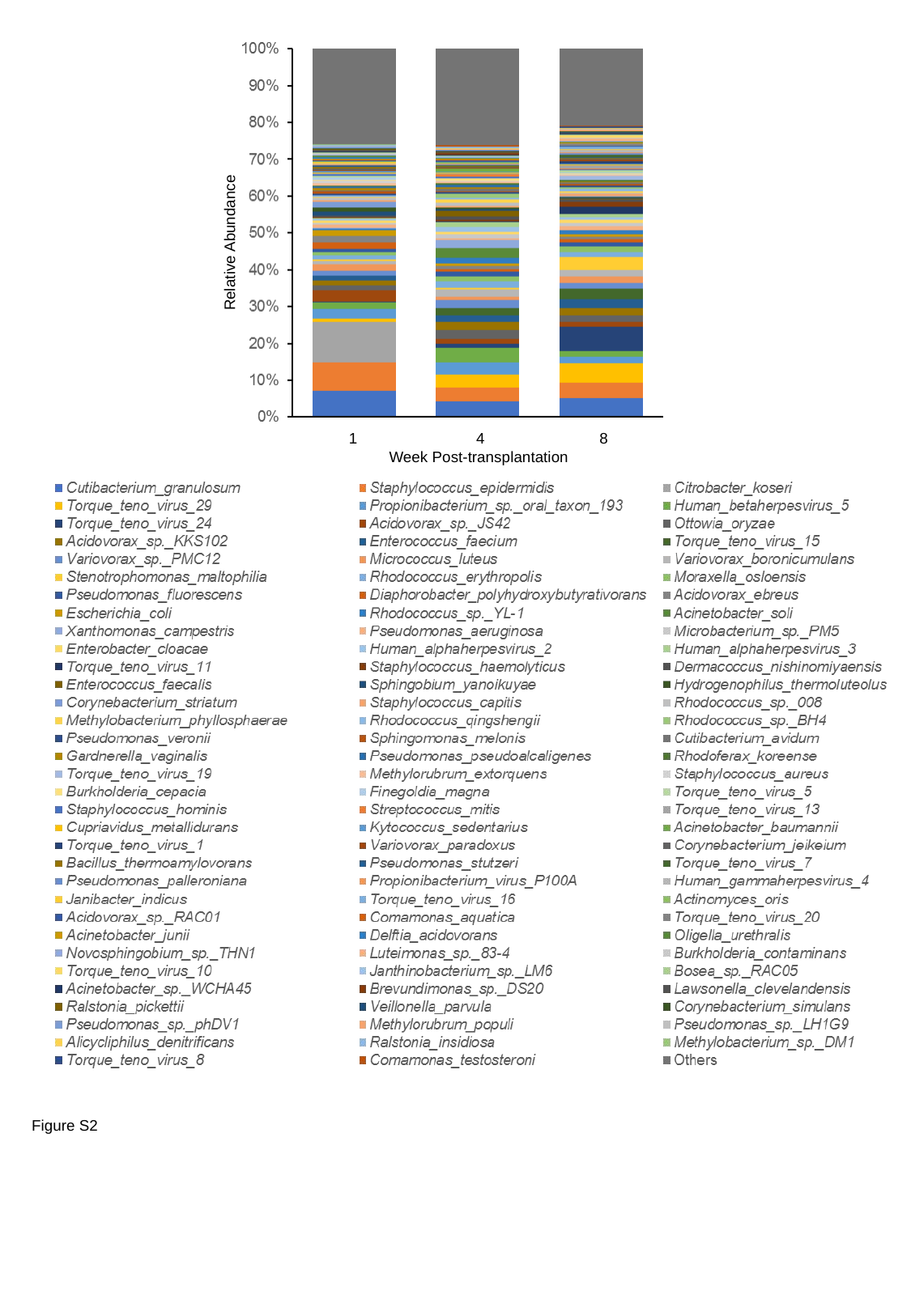

Relative Abundance
1 4 8
Week Post-transplantation
Figure S2

## Slide 3
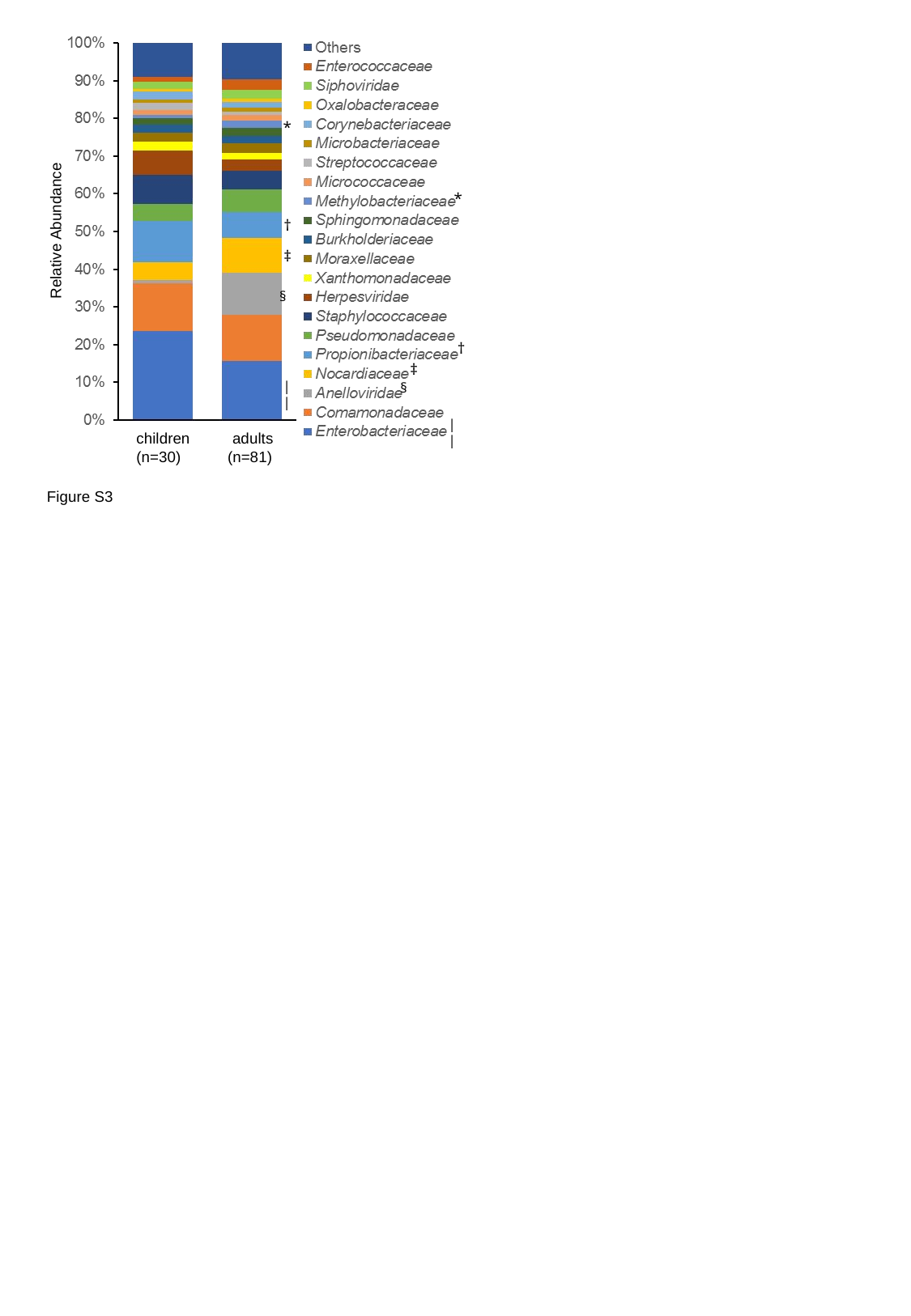

children adults
 (n=30) (n=81)
*
*
†
Relative Abundance
‡
§
†
‡
||
§
||
Figure S3
